# Effect of sonification types in upper-limb movement: a quantitative and qualitative study in hemiparetic and healthy participants

**DOI:** 10.1186/s12984-023-01248-y

**Published:** 2023-10-05

**Authors:** Iseline Peyre, Agnès Roby-Brami, Maël Segalen, Alain Giron, Baptiste Caramiaux, Véronique Marchand-Pauvert, Pascale Pradat-Diehl, Frédéric Bevilacqua

**Affiliations:** 1 UMR STMS, Ircam, CNRS, Sorbonne Université, 75004 Paris, France; 2 ISIR, CNRS UMR 7222, Inserm U1150, Sorbonne Université, 75005 Paris, France; 3 Laboratoire d’Imagerie Biomédicale (LIB), Inserm, CNRS, Sorbonne Université, 75006 Paris, France; 4 AP-HP, Hôpital Pitié-Salpêtrière, Maladies du Système Nerveux, 75013 Paris, France; 5 GRC HaMCRe, Sorbonne Université, 75013 Paris, France

**Keywords:** Movement sonification, Upper limb, Hemiparesis, Mixed methodology, Rehabilitation

## Abstract

**Background:**

Movement sonification, the use of real-time auditory feedback linked to movement parameters, have been proposed to support rehabilitation. Nevertheless, if promising results have been reported, the effect of the type of sound used has not been studied systematically. The aim of this study was to investigate in a single session the effect of different types of sonification both quantitatively and qualitatively on patients with acquired brain lesions and healthy participants.

**Methods:**

An experimental setup enabling arm sonification was developed using three different categories of sonification (direct sound modulation, musical interaction, and soundscape). Simple moving forward movements performed while sliding on a table with both arms were investigated with all participants. Quantitative analysis on the movement timing were performed considering various parameters (sound condition, affected arm and dominance, sonification categories). Qualitative analysis of semi-structured interviews were also conducted, as well as neuropsychological evaluation of music perception.

**Results:**

For both the patient and healthy groups (15 participants each), average duration for performing the arm movement is significantly longer with sonification compared to the no-sound condition (p < 0.001). Qualitative analysis of semi-structured interviews revealed different aspects of motivational and affective aspects of sonification. Most participants of both groups preferred to complete the task with sound (29 of 30 participants), and described the experience as playful (22 of 30 participants). More precisely, the soundscape (nature sounds) was the most constantly preferred (selected first by 14 of 30 participants).

**Conclusion:**

Overall, our results confirm that the sonification has an effect on the temporal execution of the movement during a single-session. Globally, sonification is welcomed by the participants, and we found convergent and differentiated appreciations of the different sonification types.

**Supplementary Information:**

The online version contains supplementary material available at 10.1186/s12984-023-01248-y.

## Background

Acquired brain lesions in adults, following stroke, head injury, or brain tumor, are major causes of acquired disability worldwide [11, 46]. These lesions induce multiple sensory, motor, and cognitive disorders. Among these disorders, motor impairments could affect 40% of patients after stroke [33]. In particular, we consider in this study the specific case of upper limb hemiparesis, characterized by impaired motor control and muscle weakness, greatly reduces autonomy in daily living activities, and thus, the long-term quality of life of patients [10].

The use of music is being studied in a wide range of rehabilitation settings [16, 39, 44, 47, 61], particularly in the case of acquired brain lesions [27, 68]. Several different methods have been investigated, such as audio-rhythmic stimulation (RAS) [65, 67], exercises with musical instruments (music-supported therapy) [1, 2, 13, 28, 52], and movement sonification devices [26, 36, 48, 58].

In this paper we focus on movement sonification, which concerns systems that enable translating in real-time motion parameters into sound or musical parameters [21, 30]. Movement sonification devices have many advantages [60]: access to a continuous 3D auditory information, fast adaptation of sound feedback to the movements performed, flexibility of use by participants with various profiles thanks to possible adaptation according to individual abilities [7, 23]. Thus, these devices present a potential added value in comparison with other sound/musical methods and tools, and offer perspectives in adequacy with the needs described in the rehabilitation framework [12, 66]. Moreover, compared to other feedback modalities such as visual feedback, the use of the auditory modality does not constrain the user's posture. In this case, the dependence to the external feedback, called the guidance effect, might be less important with auditory compared to visual feedback since sonification could encourage to focus attention on intrinsic proprioceptive information [19]. The potential interest of sonification devices as a rehabilitation support tool is therefore under investigation for different rehabilitation applications [25, 26, 37, 53, 63].

Concerning more specifically rehabilitation after acquired brain lesions, respectively in a pilot study [54] and a large-scale study [48], Schmitz et al. and Raglio et al. showed an encouraging evolution of the global dexterity scores (Box and Block Test) with sonification devices. In both situations, standard motor rehabilitation exercises were sonified.

In 2015, Scholz et al. proposed an innovative device where users learn to move in a virtual space associated with a musical scale, with the aim of playing melodies [57]. In this case, a decrease in pain scores was reported, as well as a trend towards improvement in the Stroke Impact Scale functional hand assessment scores. Nevertheless, this study did not show any improvement in scores on the other functional assessments performed (Action Research Arm Test, Box and Block Test, Nine Hole Peg Test). In a pilot study, Robertson et al. suggested that in the presence of audio feedback different results could be obtained depending on the hemispheric location of the brain lesion, and more precisely a deterioration in kinematic performances in the presence of audio feedback in the case of left hemispheric brain lesions [50].

Thus, although encouraging results have been obtained in different settings, limited functional benefits have also been reported [40]. One reason for contrasted effects could be related to the choice of sound and interaction design. In early works, the choice made was to sonify errors, by emitting “alarm” sounds when the participant does not follow the predicted trajectory model [36]. More recent approaches propose to favor the participant motivation and avoid negative reinforcement [7].

Moreover, the quality of the sound rendering has not always been a central concern, yet the choices of sound design and mapping could be fundamental to ensure the adequacy between the sound and the gesture to be performed, and thus, the effect of sonification on the movement control and learning [3, 15]. Questions about sound design and coupling modalities require further investigations [31]. In particular, these investigations must be considered with regard to the performed tasks, the user profiles they address, and individual singularities. Importantly, the need to consider multiple sonification modalities and to evaluate their effects was notably highlighted in two recent literature reviews [26, 41]. Moreover, the user perception and experience of sonification has been insufficiently studied.

In this perspective, the novelty of the present study was to evaluate, during a single session, different modalities of gesture-sound interactions, categories and types of sound feedback, with both adult patients with hemiparesis following an acquired brain lesion and healthy participants. An originality of our approach consists in using a mixed methodology, evaluating the effect of different sonification modalities both quantitatively and qualitatively. Our specific goal in this study was to measure the spontaneous effects of the sound feedback on the temporality of execution of the movement, and the subjective experience of each participant through semi-directed interviews. On the contrary to typical rehabilitation assessments where the motor task must be performed as quickly as possible (i.e. scores in assessments typically indexed on the number of objects moved [15, 17], or the number of repetitions of a movement or targets reached), we rather chose to give no instruction concerning the speed of execution of the task, and assess how the sonification could influence spontaneously the average movement speed.

## Methods

### Participants

All participants met the following inclusion criteria (Table 1): age between 18 and 80 years old, ability to understand the consent form and simple instructions, ability to answer questions during semi-structured interviews, and consent to participate.

**Table 1:** Description of the groups of participants

Status	Patients	Healthy
Gender (M/F)	6 M/9F	6M/9F
Age (mean; range)	45 years; 20–70	42 years; 21-71
Musical background (none/amateur/Pro)	8N/6A/1P	7N/6A/2P
MBEA (norms/falls)	10N/5F	15N/0F
Dominant side (R/L)	15R/0L	15R/0L
Hemiparesis (R/L)	6R/9L	/

Participants were included in the patients group if they were hospitalized in rehabilitation department of Pitié-Salpêtrière Hospital and had upper-limb hemiparesis after acquired brain lesion with sufficient recovery to initiate an elbow extension (without external help) and complete the motor task (stretch their elbow while sliding on a board). Exclusion criteria were any other neurological disease, severe neuropsychological impairments, musicogenic epilepsy, heart pacemaker, or hearing deficits requiring hearing aids.

Healthy volunteers were recruited and selected to be paired to the patient group considering sex and age range. Exclusion criteria were neurological pathology, upper-limb deficits of any origin, heart pacemaker, or hearing deficits requiring hearing aids.

### Protocol

**Fig. 1:**
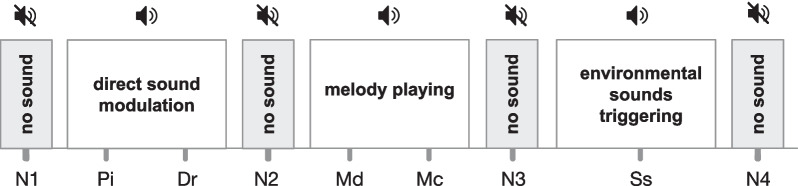
Order of the sound conditions with the “no sound” condition intertwined No sound: N1, N2, N3, N4. Sound: Pi: Pitch, Dr: Drum, Md: Discrete Melody, Mc: Continuous Melody, Ss: Soundscape. These sonification types are described in detail in section Experimental Setup

The experimental design consisted in three steps:

1. Interview and Amusia test
  An interview of each participant was carried out to evaluate their musical experience (vocal and/or instrumental education and practice), listening habits and possible hearing deficits. Three levels of musical expertise were distinguished: no musical practice, amateur experience or practice corresponding to a minimum of two years of regular vocal/instrumental training, and professional experience or practice. After the interview, their musical perception was assessed with the Montreal Battery of Evaluation of Amusia (MBEA) [45]. The participants’ scores were compared to the norm established during the validation of the tool [45] in order to identify possible deviations from the norm in each group (Chi-square test). At last, their manual dominance was assessed with the Edinburgh Handedness Inventory [42].
2. Sonification session
  During a single 1-hour sonification session the participants were instructed to extend the arm repetitively, sliding on the table with a fabric to minimize friction, following a straight trajectory. In order for the system to adapt to the motor skills of each participant, a personalized calibration was performed for each arm at the very beginning of the task to define both starting and end positions on the table that could be reached by the participant (see section Experimental setup). The instruction did not impose any particular timing to perform the movement: the participants were explicitly asked to perform the movement at the speed of their choice. This allowed us to compare average movement durations according to the participant profiles, while keeping the sound conditions order identical for all participants. During the session, participants used each arm alternatively, less-affected then affected for patients, and dominant then non-dominant for healthy subjects, with three different categories of sonification (direct sound modulation, musical interaction, and soundscape, described in detail in the section Experimental Setup) and no-sound condition. The order of presentation of the sound conditions starting on purpose from simple sound modulations, shown in Fig. 1, was identical for all participants, as we were aiming primarily to provide a comparable experience among participants. We included a no-sound condition at the beginning, the end, and between the three sonification categories, in order to assess the stability of the no sound-condition and any after-effect of each sound type on the no-sound conditions. This should allow us to ensure that the no-sound condition can be used as a participant-dependant control condition. Before each sound condition, a familiarization phase was performed to allow participants to understand each movement-sound coupling and stabilize their movement. After this phase, the participant performed ten repetitions. This number was advised by physiotherapists to avoid fatigue in patients.
3. After-session interview
  After the session of sonification a semi-structured interview of the subject experience was recorded with a dictaphone (Guide of semi-structured interview, on Additional file 1: S1). We also asked participants to sort by order of preference the sound conditions, and to choose in order 5 qualifying terms to describe their feeling in a 18 qualifier list, based on a balanced valence/arousal diagram.

### Experimental setup

**Fig. 2:**
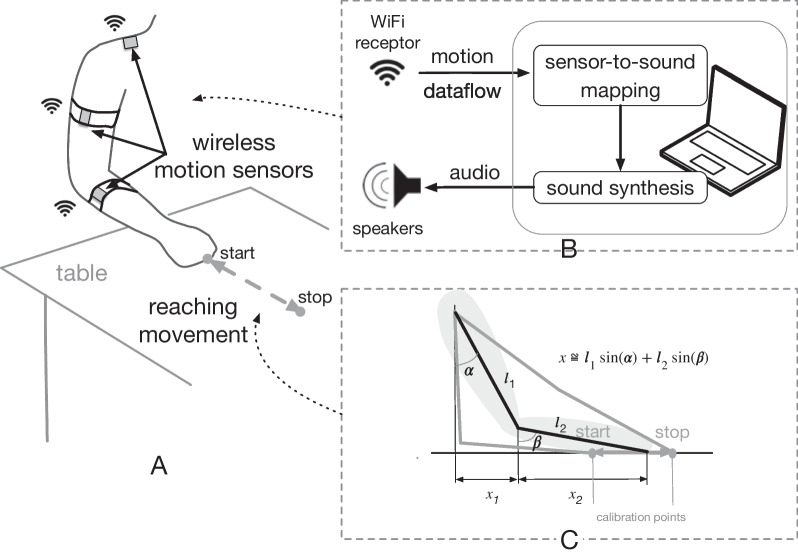
Experimental Setup. **A** placement of the motion sensors (IMU). **B** Schematic of the dataflow process and auditory output. **C** Basic description of the computation of the displacement using the two calibration points indicated as *start* and *stop* (median plane)

A specific system was built in order to provide sonification in response to the arm movement of the participant. Similarly to other recent proposed systems [4, 14, 59], we used wireless motion sensors, each containing an Inertial Measurement Units (IMU) 3D accelerometers, 3D gyroscope and 3D magnetometers, and transmits the data sampled at 200 Hz in real-time through WiFi. Precisely, three motion sensors were attached to both arms, as depicted in Fig. 2. The transmitted data include the orientation of the IMU units (i.e. the Euler angles), which are used to compute a normalized displacement parameter after performing a calibration consisting in recording the IMUs data at the start and stop positions (indicated in Fig. 2A). This displacement parameter is used as the input parameter in the sonification system (described in Section Sonification strategies). As mentioned in the protocol, the calibration procedure was performed at the beginning of each participant's session, which allows for adapting the sonification to each participant's motor capabilities, since the actual arm displacement can be different for each one.

The laptop computer, connected to a soundcard and speakers, operates the sonification using a program written with Max7 (Cycling’74) and the extension MuBu for Max [56]. This library allows for performing data signal processing, and controlled sound synthesis. The software is also used to record all the IMUs data to the computer, along with the sounds. The audio rendering system was composed of one stereo speaker in front and two additional mono speakers in the back of the participant in order to create an immersive sound environment.

The displacement data, along with the raw IMUs data and audio output were recorded during all movement cycles, and saved in the computer. Video recordings were also performed during all the experiments, allowing the verification of the data collected with the IMUs.

**Fig. 3:**
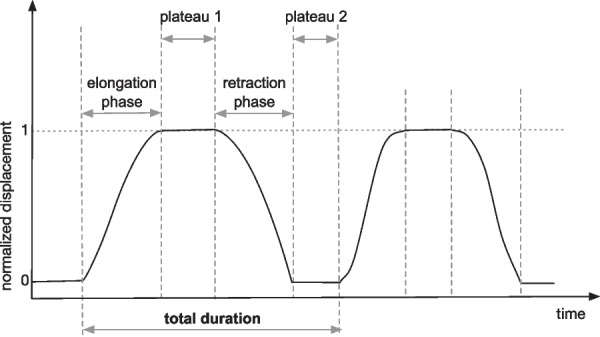
Normalized displacement over time with the different computed phases. **1** The “elongation phase” of the upper-limb (extension of the elbow), **2** The “plateau-1”: phase of maintenance in a position of maximum upper-limb elongation, **3** the “retraction phase”, return to the initial position, **4** the “plateau-2” phase in the initial retracted position, elbow bent, before initiating a new extension-flexion cycle

A data analysis script (Matlab, R2018a, Mathworks USA) was developed allowing for data visualization, and semi-automatic segmentation of the displacement data. From this, we computed the total duration time for each movement cycle (see Fig. 3). This led us to compute 5 different time duration: extension duration, plateau-1 duration, return duration, plateau-2 duration, and total duration time (sum of the 4 previous ones).

### Sonification design

We decided to implement 5 different types of sound, classified in 3 different sonification categories, in order to evaluate how different sounds and musical interactions could influence the movement timing and how they were perceived by the users in this context. Audios and sound spectrograms are presented in Additional file 1: S2.

1. Direct sound modulation
  This category of sonification has been largely implemented in sonification systems and reported in the literature [18, 41].
  Pitch (Pi): direct relationship between the reaching distance and the pitch. In order to avoid the annoyance of a pure tone, we use granular synthesis in order to vary the pitch of a sample sound that contains a rich spectrum (from 92 to 500 Hz). The farther the reaching point, the higher the pitch. The range of variation of the fundamental frequency is from 92 to 122 Hz, with a strong harmonic varying from 279 to 376 Hz.
  Drum (Dr): direct relationship between the reaching distance and the tempo of a regular beat pulsation. We used a drum sound, with a regular rhythmic pattern (such as 4 eighth notes). The farther the reaching point, the faster the tempo. The range of variation is from 3.2 to 16 Hz beats. At the fast tempo, the drum sounds like a drum roll.
2. Melody playing
  Sonification implying music can potentially be motivating for the participant as shown previously [26]. In this paradigm, the user can play a melody by moving the arm. All the notes are programmed, so the task consists in activating the progression of the melody. Two distinct cases were implemented:
  Music/Discrete (Md): a full forward arm movement triggers a “discrete melody”, following a tonal harmonic progression (based on Concerto No. 5 in F Minor, BWV 1056). The movement triggers a different part of the melody at each outward and backward movements (four notes per outward or backward). This sonification was previously used in a music education scenario [29].
  Music/continuous (Mc): a full arm movement enables one to continuously “play” a complete musical phrase, using the so-called gesture follower technique, which has been used in music pedagogy [5, 6]. In this system, a time progression index of the gesture is estimated by comparing the performed gesture with a displacement profile recorded previously. Then, this estimated time progression index is used to trigger notes of the melody. The piece was a record of the Prelude in C Major by J.S. Bach interpreted by Glenn Gould.
3. Environmental sounds triggering
  This sonification category is based on everyday listening, invoking recognizable sound environments [34]. In this paradigm, the reaching movement is divided in three different zones, each one being associated with a specific environmental sound, called ‘soundscapes’.
  Soundscape (Ss): The reaching movement is divided in three equal parts. Each one triggers, respectively, sounds of wind, river and birds.

## Data analysis

### Movement analysis

The statistical analyses were performed with JMP software^®^ (SAS Inc., Cary, NC, USA). All tests were 2-sided. A p-value ≤ 0.05 was considered statistically significant.

A first step consisted in testing the homogeneity of the “no sound” conditions using analysis of variance (ANOVA) on repeated measures (or Friedman non parametric test on ranks when underlying assumptions were not verified), to evaluate any order effect in the no-sound sequences. Differences between these sequences being non significant, data were normalized by dividing duration values by the average of the no sound values in order to take into account inter-individual variability. Importantly, the normalization is performed for each arm, considering always identical distances between sound and no sound durations. The *normalized time* is thus equivalent to a *ratio* between sound and no sound durations (which could be alternatively expressed in %). Then, for each participant (patient or healthy participants) and arm considered, Student paired t test (or Wilcoxon signed-rank test), Anova on repeated measures (or Friedman test) and mixed linear models were performed on normalized data taking into account sound context. This was followed, when needed, by post-hoc Tukey HSD analysis (or Durbin-Conover test). When parametric tests were applied we made sure that the underlying assumptions (normality, homoscedasticity or sphericity for repeated measures) were valid.

### Participant’s experience analysis

In order to obtain the average preference of sound and qualifying terms used by the participants, we associated 1 to 5 points (5 being the preferred) to each sound or qualifying term for each individual ranking order. We then calculated the average points for each sound or terms.

The audio recording of each semi-structured interview has been transcribed verbatim. Three experimenters (IP, BC, FB) carried out the thematic analysis of the transcribed interviews [8]. Each experimenter read the transcription and generated individual codings from the participant’s interviews. The experimenters then gathered the codes and kept the common codes or the ones that may not be common to the three experimenters, but that reached a consensus after discussions. From the selected codes, we defined thematic axes, and we kept a list of illustrative quotations for each axis. For each result we distinguish particularities of each group of participants (designed as “P” concerning patients, and “H” concerning healthy participants).

## Results

### Participants description

Two groups of subjects participated and followed the protocol entirely: 15 patients with motor deficit (hemiparesia) resulting from acquired brain lesions, and 15 healthy participants were included.

Descriptive information about gender, age, musical background, MBEA scores, and side of hemiparesia are reported in Table 1. More details about each participant are reported on Additional file 1: S3 (Descriptive data of participant’s profiles).

All participants were right-handed, and gender repartition, age and musical background are similar between groups. Motor impairments were evaluated when patients were admitted to the hospital, and were not reliable at the time of the inclusion in the experiment. For time constraints, it was not possible to perform other motor assessments before the experiment. The design of the normalization procedure was aimed at reducing the influence of the different levels of motor impairments. This point will be again specified in the results, discussion and conclusion.

Comparative analysis of MBEA scores revealed lower scores in patient groups than in healthy ones (Additional file 1: S4). This difference is at the limit of significance (test Chi^2^ p = 0.05).

### Movement data results

#### Individual data

**Fig. 4:**
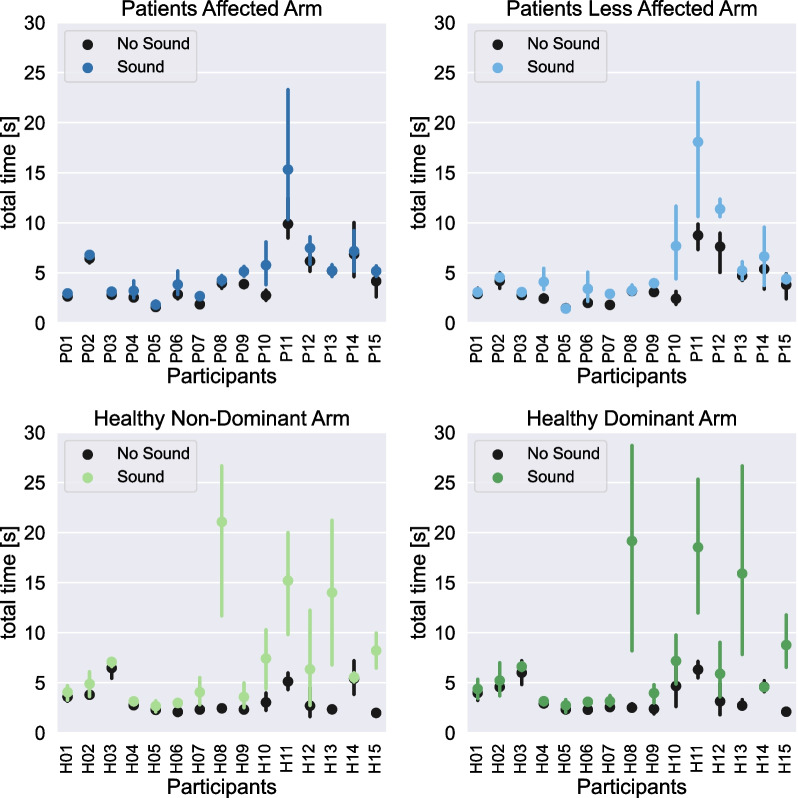
No sound vs sound condition, for patients and healthy participants. The error bars correspond to the 95% confidence intervals

Data without normalization of the averages of the total durations performed by each individual with no sound (N1, N2, N3 and N4) and with sonification (Pi, Dr, Md, Mc, Ss), are presented in Fig. 4, considering the subject group (patient and healthy participants) and the arm (paretic side vs. less affected, and dominant vs. non-dominant).

In Fig. 4, it appears that the average duration of a complete cycle varies across participants. This result must be considered in regards to the fact that we did not give any timing constraint on the movement performance. This is also probably linked to different motor ability, especially in the patients with different levels of motor impairment.

We also observe that there are more variations in the sound conditions compared to the no sound conditions. More precisely, as shown in Additional file 1: S5, the four “no sound” conditions were compared for all participants, and no significant difference was found. Therefore, this stability confirmed that the “no sound” condition can be used for normalizing each participant's sound conditions measurement.

#### Comparison between the sound and no sound conditions

**Fig. 5:**
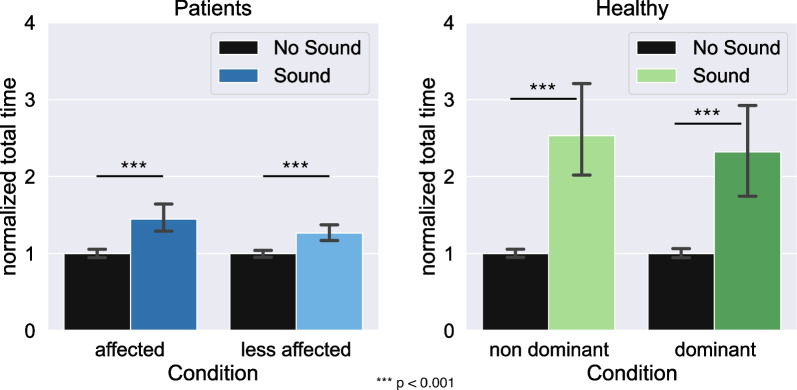
Mean for the patients (left) and healthy participants (right), for all the sound and no sound conditions, considering the different arms (affected / less affected for the patients, and non-dominant/dominant for the healthy participants). The error bars correspond to the 95% confidence intervals

The comparative analysis of the average of total cycle durations with no sound compared to sonification shows a significant difference for the normalized total time, for each situation considered (p < 0.001 for both arms in patients and healthy participants) (Fig. 5). Specifically, the total average duration increased with sonification compared to cases without sound feedback.

If we consider in more details the duration of the different phases constituting the extension-flexion cycles of the elbow, we observe significant differences in the average duration of plateau-1 (phase of maximum elongation), retraction phase, and plateau-2 (phase of minimum elongation between two extension phases), when comparing sonification to no sound conditions for both groups. Plateau-1 and the return phase were longer whatever the arm considered both in patients and healthy participants (p < 0.001). Thus, participants remained in maximal extension for a longer time with sonification compared to no sound condition and returned to the starting point slower in the presence of sonification compared to the no sound condition. The average duration of plateau-2 were also longer both in patients for paretic and less-affected arm (p < 0.05 and p < 0.001, respectively) and in healthy participants for non-dominant and dominant arm (p < 0.005 and p < 0.05, respectively).

Concerning the extension phase, significant differences between the sonification and no sound conditions were observed in the healthy participants for both arms and in patients for the less affected arm (p < 0.001).

#### Comparison between each sound condition

The comparative analysis of the average of total cycle durations according to the sound feedback categories (Anova on repeated measures) shows significant differences between sonifications categories, in the case of the paretic arms of patients and the dominant arms of healthy participants (p < 0.05) (Additional file 1: S6). These significant differences are not found in the other situations (less affected arms of patients and non-dominant arms of controls).

### Results concerning the participant’s experience

#### Sound preferences and Experience qualifiers

We report the individual rankings, rated from 1 to 5 points, according to the hierarchical preference of the sound feedback (Table 2) and the qualifying terms respectively (Table 3). The most appreciated sound feedback by both groups of participants is the soundscape, followed by the continuous music. Among the 18 qualifiers list, *playful* is the first shared term for both groups of participants to qualify their experience.

**Table 2:** Sound feedback rankings

Patients	Healthy
Soundscape (59/75)	Soundscape (57/75)
Continuous music (54/75)	Continuous music (51/75)
Discrete music (35/75)	Pitch (42/75)
Pitch/drum (31/75)	Discrete music (39/75)
	Drum (34/75)

Scores could range from 15 (if the sound feedback was ranked last by each participant) to 75 (if the sound feedback was ranked first by each participant)

**Table 3:** Experience’s qualifiers rankings

Patients	Healthy
Playful (30/75)	Playful (48/75)
Surprising (28/75)	Amusing (31/75)
Stimulating (27/75)	Captivating (28/75)
Intuitive (26/75)	Stimulating/intuitive (23/75)
Amusing (23/75)	

Scores could range from 0 (if the qualifier was not chosen from the list) to 75 (if the qualifier was ranked first by each participant)

#### Thematic analysis based on semi-directive interviews

The thematic analysis revealed six common themes across subject groups.

##### Reported feelings using the system:

First, the majority of participants reported feeling a difference in the sound context when performing the task (24/30; 14 Patients—10 Healthy), and they preferred performing the gestures in the presence of sonification (29/30; 14P-15H).

Second, the task performance was not considered to be more difficult with sonification than without in the majority of cases (22/30; 11P-11H). Thirteen participants (7P-6H) even reported that it was easier to perform the forearm extension task with sonification. Nevertheless, six participants (2P-4H) reported the experience being occasionally more difficult with some specific couplings, which they justify with two different reasons: a mismatch between the sound type and the gesture to be performed (Md), and when a specific movement quality was required for the sound production (Mc). Concerning the first aspect, several participants underlined that the jerky sound of the discontinuous melody was not matching with the representation of a regular gesture to be carried out. That induced a desire to adapt the gesture in relation to the produced sound, which implied then to perform it in a jerky way. Concerning the second aspect, the participants specified that Mc generates an expectation for the quality of the music produced. In turn, this would require a finer motor control (H01): "Since it’s music, I want it to sound like something fluid that one could listen to".

Finally, one participant mentioned a notable distinction between perception and volition (P04): "The impressions were not different but the intentions could be". This participant reported a deeper involvement in the task performance in the presence of the sonification: "[…] The movement is more voluntary when it produces a sound".

##### Sound as cues:

Looking more specifically at the interaction between movement parameters and sound coupling, the participants spontaneously mentioned a notion of “cues”, as guides for the movement. This appeared recurrently while mentioning various movement characteristics: amplitude, fluidity, regularity and reproducibility.

Among the characteristics mentioned, temporal aspects were very predominant. Several types of the sonifications effects were described with respect to the temporal characteristics of movement and sound, such as a modulation of the feeling of time (P07): "I had the impression that when I perform a movement with the sound I took more time, I went less quickly to do it", the temporal reference mark (P09): "With music we have a reference point, we keep the same cruising pace", or the more conscious search for an adaptation to the representation conveyed by the sound, in order to obtain a certain sound quality (H08): "When there was no sound I always performed at the same speed, when there was sound I varied the speeds a little because I wanted it to fit with the sound".

##### Perceived interaction modalities:

For a minority of participants, the interaction modality was unidirectional: four of them felt that the sounds led the movement (4 participants, 3P-1H), and four other participants felt that the gesture controlled the sounds (or vice versa that the sounds followed the gestures, 3P-1H). Other participants (4 patients) expressed having experienced a feedback loop. According to them, the gestures triggered the sounds which in turn provided them with feedback on the gestures, allowing them to adapt to the perceived sound/music.

Finally, for the majority of the participants, the experience of the interaction varied and evolved during the experiment (18 participants; 5P-13H) according to 3 main parameters: the type of sound feedback (1P-6H), the arm performing the task (3P), and the evolution of their understanding of the functioning of the system during the experiment (3H). Regarding the categories, types of sound feedback, and the proposed couplings, participants unanimously expressed that the gestures controlled the sounds for the simple couplings (Pitch and Drum) while the gestures adapted to the sounds for the musical couplings, especially the continuous melody (Mc). Regarding the way the arm performing the task affects the experience of the interaction, patients specified that, for the paretic arm, the gesture controlled the sound, whereas, with the less affected arm, the sound controlled the gesture, or that the gesture adapted to the sounds. Finally, regarding the evolution during the experiment of the interaction understanding, participants expressed that they followed the sound at first, and that later they voluntarily controlled their gesture in order to modulate the sound. H13: "At the beginning I had the impression that I was trying to follow the sound…well to make a gesture following the rhythm, and then I understood that I could control the sound myself with the gesture".

All of these findings suggest that the nature of the sound feedback and the coupling modalities had an influence on the perception of the interaction and on the participants’ experience. H06: “The coupling between the sound and the movement changes the experience of the movement, and so even if you’re trying to do the same movement, even if it’s exactly the same movement, the way you experience it is different, the involvement of the person in the task is really changed”.

##### Reported emotions

Some participants spontaneously stated that the task was more enjoyable, funnier, more engaging, and more interesting with any type of sonification. Four main affective states were expressed by the participants: playfulness, curiosity, frustration and relaxation. The notion of playfulness is predominant in the spontaneous comments of the participants (8P-9H). Many participants also mentioned their curiosity and surprise at discovering the device. This surprise was often at the origin of the playfulness mentioned above. P07: "I was surprised by the sounds I was making when I was doing the acceleration and deceleration movements. It surprised me, and I liked it, I found it very playful". In other cases, the curiosity was formalized by expecting something from the device. Frustration could also emerge in reaction to the restrictive framework of the instruction: H08: “The fact that I could only do one movement of extension of the arm is a little frustrating because I would have done other movements […] me in any case I wanted to adapt my movements to the sounds”. Finally, the notion of relaxation was expressed many times by the participants, more particularly regarding two couplings: the 'continuous melody' and the ‘soundscape’, implying in some cases body feeling and the task performance. P09: “With the music it softens, it soothes, it’s like we were being massaged, as if we were being put in a second state to be willing. At one point there was music with the sea, the wind, it relaxes you, when you are obliged to make a movement and you can't do it, it relaxes you”.

##### Mental imagery

Many participants associated the gesture-sound couplings with different mental imagery. The ‘pitch’ was associated with images of a vinyl record, a soft car engine, an ocean or even described as celestial. The 'drum' has been associated with muffled hammering or African drums. The 'discontinuous melody' has been the object of less and contrasted associations (mandolin, stalactic in a cave), although images of bouncing movements have been widely mentioned. The 'continuous melody', which original musical piece was sometimes recognized and named, was associated with the idea of spring, and 'dream-space'. This sonic coupling, in some cases, created the illusion of being a musician (H13): 'I caught myself for thirty seconds as if I were Mozart, so I was very pleased with myself'. Finally, the 'soundscape', a metaphorical space by design, was the most prolific in terms of images, very often associated with the idea of escaping. H09: "There were images that appeared, […] I imagined a kind of walk in a forest, we walk next to the river, then we arrive in a meadow, where there are birds… we imagine the scenery that goes with it".

The stimulation of mental imagery is linked to the participants' preferences: the more the person appreciates the coupling, the more his or her mental imagery is triggered and stimulated. P08: "Every time there was music, I imagined a scene or a moment that I experienced. Especially on the music that I liked in fact ".

##### Attentional modulation

Evocating the feeling of escape, as well as various emotions, led several participants to report having felt a modulation of their attention during the task, and this depending on the sound context. However, differences across groups should be highlighted.

In the control group, the majority of participants mentioned that their attention was mainly focused on the sounds (10H) H01: "When there was a sound I was thinking less about the movement, I was thinking less about reaching out, I was focusing on the sound". For the other five participants in this group, they could either focus their attention simultaneously or alternately on the sound and the gestures. One participant specified the effects of the feedback loop on their attention and evoked the notion of embodiment: "The attention is not on the movement itself, but on the movement in the context of the effects it has on the music, so I think it changes a lot our way of thinking about the body during the movement".

Within the group of patients, the comments were more contrasted: 4 expressed that their attention was rather focused on the sounds, 4 rather on the gestures, while the others mentioned that the focus of the attention varied, either according to their appreciation of the coupling, or according to the arm performing the task. Indeed, as the gesture could be difficult to perform with their paretic arm, the attention could then shift to the gesture, while being supported by the sound: P06: "[affected side] we are very preoccupied by the very basic movement we have to do. The extension is difficult so we focus on the movement. When I liked the sounds, the attention was directed to the sounds".

## Discussion

### Temporality: with sound versus without sound feedback

Our goal was to evaluate the spontaneous effect of sonification on temporal parameters and user experience, without giving any temporal constraints to the participants, as stated in the instruction that we provided where the gesture timing was left free.

The results we obtained suggest that, whatever the status of the participants (patients with sequelae of an acquired brain injury, or healthy participants) and the arm considered (paretic vs. less affected or dominant vs. non-dominant), the presence of a sound feedback has an effect on the participant’s feeling during the experience and on the gesture performance timing of the extension-retraction of the elbow on a table.

While we observed large inter-individual differences, partially due to heterogeneous motor ability and/or impairments, the normalization procedure enabled us to highlight a significant global slowing down of the movement in the presence of sound feedback, with in particular a longer duration of maintenance in maximum extension and minimal extension, and a slowing down of the return phase in all the situations considered. This observation of a longer duration of maximum extension in the presence of sound feedback is encouraging as to the possibility of using it in a rehabilitative context, in order to prolong the duration of a posture maintenance during stretching exercises, with the aim of promoting a progressive gain in the amplitude of movement.

Different mechanisms that could shed light on the reasons for the differences in temporality of movement in the presence of sound feedback can be considered, regarding the participants' comments and the literature.

A first hypothesis would be that the induced attentional load may have contributed to the slowing down of the movement in the presence of sound feedback. However, this hypothesis does not seem to be in agreement with the analysis of the qualitative results and the literature. Indeed, the participants indicated that the sound feedback worked as cues, allowing them to pace the movement, and this even when no tempo or intrinsic rhythmic element was present in the sound feedback (cases of the pitch and the soundscape). The presence of sound feedback can suggest an implicit timing. For example, in the case of the continuous musical sound feedback (Mc), although the speed remained free, the implicit tempo of the piece could suggest a movement speed. In this regard, Sihvonen et al. [61] suggests that in the presence of sound feedback participants make inferences about the timing of sound events, consequently influencing the temporality of movement completion. The repetition of a movement at a regular and constant tempo with audio feedback would thus be likely to induce its automation, and the attentional system could be less solicited thanks to this temporal cueing function. Further, research on attentional processes mobilized during motor learning has shown that external focus induces a more automatic control, less costly, and therefore beneficial for the realization of the movement [24]. From this perspective, assuming that sound feedback are sources of external focus and implicit learning, their use should therefore allow for limiting attentional load, provided that the design is adapted [19]. Also, the observed slowing of movement would therefore not be attributable to an attentional overhead. Nevertheless, in the case of our experiment, as this was the first use of a motion sonification device for all participants (both healthy and patients), it cannot be totally excluded that other processes were involved. In particular, it is more usual for novices to adopt a strategy of attentional focus on internal parameters and explicit learning [24]. Although the intrinsic principle of sonification devices is conducive to external focus and implicit learning [19], the attentional processes mobilized during the use of sonification devices remain insufficiently known to date. In particular, it would be necessary to study the strategies used according to the users' experience in order to determine in which cases sound feedback can be considered as distractors [35, 43], sources of external focus, or even sources of internal focus if we consider that an optimized mapping could be likely to favor attention to proprioception. In the case of rehabilitation, it is commonly accepted that it is important to limit attentional distractors and that dual-task situations can be too costly and diminish motor performance in the case of gait [38]. However, decentering participants' attention during the execution of a motor task can, under certain conditions, also improve its completion [32]. In this perspective, investigations centered on the mobilization of the attention aroused by sonification in the case of rehabilitation should be carried out. On this topic, the comments of the participants in our study suggest differences in strategies between individuals. Some participants mentioned focusing on the sound source, others focusing on the movement, or an oscillation in the source of attentional focus, navigating between internal (movement) and external focus (sound), depending in particular on the sound feedback used, or even in some cases a joint attention to the different sources.

A complementary approach corresponds to considering sound feedback as information contributing to internal models of movement control. Based on work on motor control and learning [21, 22], proposed to consider motion sonification from the perspective of multisensory integration theory. Under a reserve of few conditions (design adequacy and sound mapping) the effects of motion sonification would not be solely related to rhythmic adaptation. Building on the work of Rauschecker [49] Effenberg et al. [23] and Schmitz et al. [55] clarified that, in a manner comparable to the processing of visual information, two dissociated pathways for the processing of auditory perceptual information should be considered: the conscious ventral pathway (“what”) and the unconscious dorsal pathway (“where”). According to these authors, the dorsal pathway, which is unconscious and particularly important for motor control, could be brought into play during the sonification of the movement according to the design and the sound mapping. Thus, the auditory information related to the movement transmitted during the sonification would contribute to the improvement of the sensorimotor representations, and to the internal models, by being processed at a non-conscious level.

In the case of our study, the participants reported that, beyond an impression of modulation of the movement speed, the sound feedback exerted a more global influence on their volition, their intention and their implication in the movement. We could therefore suppose that a conscious processing also took place during the task performance, along with a modulation of the sense of agency. Beyond an effect on the physiological parameters of the movement, the movement sonification was shown to possibly modify the participants’ body perception and representation [62]. By extension, movement sonification could therefore modify their relationship to their movement by diverting them from a functional goal to an aesthetic one [64]. In our study, the considered task does not involve a functional goal as in the case of pointing or grasping an object. The presence of a sound feedback thus provides the participant with a goal for the task, allowing the transition from a simple repetition task to a goal-oriented task, we can refer to a “sound-oriented” goal [7]. The presence of a sound feedback thus modifies the intentionality of the gesture. Moreover, the interactive process influences the participants’ perception of their movement control, and allows them to playfully experiment situations, alternating between sensations of producing or following sounds. In this perspective, this modulation of the sense of agency in the presence of sound feedback, especially reinforced during the first use of a sonification device, could also explain the global slowing down of the movement.

In order to shed light on the processes (neurophysiological, perceptual, attentional and cognitive) involved in the execution of simple gestures with a movement sonification device, further studies are necessary.

### Specificity of the sound feedback

The comparative analysis of the temporal movement data reveals significant differences according to the category of sound considered in the cases of paretic arms of patients and dominant arms of healthy participants. No significant differences were found in the other cases, and concerning comparative analysis of each type of sound condition. However, similarities of distribution profiles encourage deepening investigations, considering our small sample size (15 participants in each group) induced a low statistical power. Also, it would be necessary to randomize the order of presentation of sound feedback to further describe a possible differentiating effect of each sound. In addition, very contrasting feelings according to the types of sound feedback were expressed in the participants' interviews and also support the interest to investigate further the effect of each sound feedback on movement timing.

The temporal dimension of a sound feedback could indeed influence the movement performance differently. In this study, Drum displays explicit timing information (direct variation of a pulse according to the extension of the elbow) while discrete and continuous music (Md, Mc) displays implicit timing information. By implicit timing information, we refer to cases where the participants try to adapt their movement to render the musical extract as they anticipate, using prior knowledge. The intrinsic temporal and aesthetic sonification characteristics seems to influence the movement performance timing and feeling, which was previously reported in the literature [19]. In particular it seems that a higher musical quality, as in the case of the continuous melody, was perceived as a motivational added value, even if it imposes larger constraints on the movement performance.

The participants' interviews also pointed towards the notion of affordance. In the case of sounds, affordance can be defined as the opportunities for actions elicited by a sound [20], in other words, the sound characteristics eliciting a representation of an associated movement [9] . In our case, the discontinuous sound feedback (Md) was indeed associated with the desire to perform bouncing motions, rather than a continuous sliding motion. These remarks underline the intrinsic link between representations of movements associated with sound feedback and the necessity to take them into consideration during the sound design for a correct adequacy between the characteristics of the proposed sound and the motor task to be performed. It is very interesting to note that, beyond the category of sound feedback, the specific characteristics of each sound feedback are likely to influence the participants’ feelings and emotional states. Precisely, the specificities of each “musical” condition induce different feelings and a notable preference. These observations are in line with the literature [19, 51] and therefore support the hypothesis that the nature of the sound feedback used, its characteristics, the sound design, and the coupling modalities, influence the movement timing and participant’s experience.

## Conclusion, limitations and perspectives

In conclusion, the sonification has a significant effect on the temporal execution of the movement during a single-session. This effect was established for both healthy participants and patients with upper-limb hemiparesia after acquired brain lesion. Moreover, qualitative analysis pointed out that performing the task with sonification changes participants' feelings, notably concerning intentionality, volition and motivation during movement.

Specificities and intrinsic characteristics of each type of sound feedback and gesture-sound coupling could be likely to influence the effect of sonification on the temporality of the gesture and its experience. The majority of participants have a preference for the soundscape and musical feedback, under reserve of congruence with the gesture to perform. Special attention must be paid to the potential difficulty induced and the emotions likely to be felt. The qualitative results suggest that it would be interesting to investigate attentional processes mobilized by the sonification modalities with multiple motor tasks and various participants profiles, and the possible evolution of the attentional cost according to the training.

Beyond the limitations present in this study (limited sample, motor impairments heterogeneity, single session without follow-up over several sessions, no randomization of the order of presentation of sound feedback, focus on the temporal criterion in the analysis of movement data), it contributes to current open methodological questions concerning the evaluation of movement sonification, specifically in the context of rehabilitation.

This study calls for further investigations. A first question concerns the most efficient use of sonification, whether an immediate and spontaneous effect on movement performance with sound is finally preferable to the results of progressive learning with long-term training. Second, it remains to better establish relevant parameters or criteria (physiological, functional, attentional, motivational) for the evaluation of the effectiveness of a movement sonification system. Third, it seems important to evaluate any potential unconscious effect of the sonification of movement on voluntary motor skills.

### Supplementary Information

**Additional file 1:** **S1.** Guide of semi-structured interview. **S2.** Sound Spectrogram. **S3.** Descriptive Data of Participant’s Profiles. **S4.** Amusia Scores. **S5.** No Sound Serie. **S6.** Sonification Categories results. **S7.** Sound condition results.

## Data Availability

The data sets and/or analyzed during the current study are available from the corresponding author on reasonable request.

## Abbreviations

MBEA: Montreal Battery of Evaluation of Amusia
IMU: Inertial Measurement Unit
N: No Sound
Pi: Pitch
Dr: Drum
Md: Music discrete
Mc: Music continuous
Ss: Soundscape
P: Patients
H: Healthy participants
ANOVA: Analysis of Variance
