# Supplementary material for: Effect of sonification types in upper-limb movement: a quantitative and qualitative study in hemiparetic and healthy participants
Source: J Neuroeng Rehabil. 2023 Oct 5;20:136. doi: 10.1186/s12984-023-01248-y (PMC10552218; doi:10.1186/s12984-023-01248-y)
Supplement: Supplementary file 1 — Additional file 1: S1. Guide of semi-structured interview. S2. Sound Spectrogram. S3. Descriptive Data of Participant’s Profiles. S4. Amusia Scores. S5. No Sound Serie. S6. Sonification Categories results. S7. Sound condition results. [file 12984_2023_1248_MOESM1_ESM.docx]

# Additional file

## S1: Guide of semi-structured interview

1. Could you summarize the study (steps, elements you noticed, those you appreciated and didn’t like)?

2. Could you describe your impressions, physical feelings, and the thoughts that crossed your mind when you carried out the different stages of the experience?

3. Did you notice any differences according to the sound context when you were doing the movement? Which differences? In which cases?

4. During gesture-sound coupling, on which elements did you focus your attention?

5. a. What sound contexts did you appreciate? For what reasons?

b. What sound contexts did you not appreciate? For what reasons?

c. Could you rank the sound contexts in order of your preference?

6. Answer only for patients: Would you use this type of device to continue your rehabilitation at home?

7. In this list, choose the terms that correspond to your feelings during the experience. Then, rank them from 1 to 5.

Unpleasant

Uncomfortable

Intuitive

Surprising

Irritating

Difficult

Tiring

Playful

Captivating

Relaxing

Easy

Embarrassing

Stressful

Stimulating

Annoying

Frustrating

Pleasant

Amusing

## S2: Sound Spectrogram

##
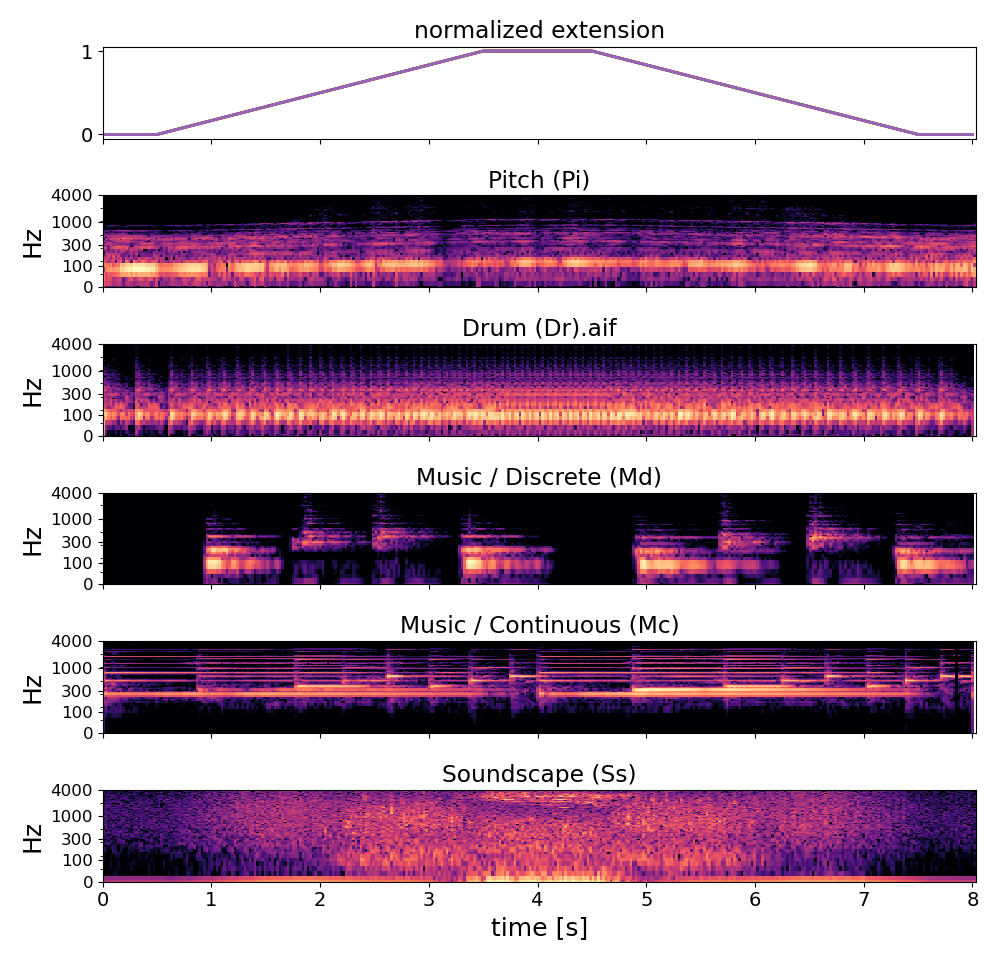


Sound spectrogram for each sound condition. This was measured using the same simulated extension for each sound condition to enable the comparison. This was performed using the Librosa library using an FFT window of 2048 pts and a hope length of 1024 pts, at sampling frequency of 44.1 kHz.

.

## S3: Descriptive Data of Participant’s Profiles

Fifteen participants with subacute or chronic hemiparesis (1-16 months) due to a cerebral injury were recruited from the rehabilitation department of Pitié-Salpêtrière Hospital. They were inpatients, aged between 18-80 years old, primarily hospitalized for a motor deficit (hemiparesis). The causal injury was in most of the cases (11 patients) due to an ischemic stroke, in the territory of the middle cerebral artery in 8 patients. Three patients suffered from hemorrhagic stroke and the last one from a traumatic brain injury (Table S3.1). None of them had major cognitive or perceptual impairments that would limit participation in the experiment. They were able to understand the consent form and simple instructions, and to answer questions during semi-structured interviews, and they consented to participate.

| **Patient** | **Age** | **Gender** | **Type** | **Site** | **Paretic Arm** | **Time between lesion and sonif session** (months) | **Musical Background** | **MBEA Average** | **Hearing troubles / Exploration** |
| --- | --- | --- | --- | --- | --- | --- | --- | --- | --- |
| P1 | 34 | F | Hemorrhagic Stroke | Right Frontal Lobe | Left | 2 | Amateur | 25.8 | None |
| P2 | 42 | F | Ischemic Stroke | Pons-Midbrain | Right | 4 | None | 23.4 | Tinnitus |
| P3 | 49 | M | Ischemic Stroke | Territory of Left Middle Cerebral Artery | Right | 1 | None | 27 | None |
| P4 | 27 | M | TBI with post-TBI hematoma | Right Fronto-Parietal Lobe | Left | 9 | Amateur | 19.2 | Tinnitus |
| P5 | 40 | F | Ischemic Stroke | Territory of Right Middle Cerebral Artery | Left | 4 | None | 17.2 | None |
| P6 | 59 | F | Ischemic Stroke | Territory of Left Middle Cerebral Artery | Right | 1 | Amateur | 22.4 | None |
| P7 | 45 | F | Ischemic Stroke | Territory of Left Middle Cerebral Artery | Right | 7 | None | 23.6 | Audio test OK |
| P8 | 28 | F | Ischemic Stroke | Territory of Right Middle Cerebral Artery | Left | 10 | Amateur | 24.2 | Audio test OK |
| P9 | 47 | M | Ischemic Stroke | Territory of Left Middle Cerebral Artery | Right | 16 | None | 21.2 | Audio test OK |
| P10 | 55 | F | Hemorrhagic Stroke | Pons | Left | 13 | None | 21.4 | Audio test OK |
| P11 | 70 | F | Hemorrhagic Stroke | Right Capsulo-thalamus | Left | 3 | Amateur | 26 | None |
| P12 | 64 | M | Ischemic Stroke | Right Paramedian Pons | Left | 1 | Professional | 26.6 | Transient Tinnitus Audio test OK |
| P13 | 20 | F | Ischemic Stroke | Right Anterior Cerebral Artery | Left | 2 | None | 26.4 | Transient Tinnitus Audio test OK |
| P14 | 42 | M | Ischemic Stroke | Left Posterior Cerebral Artery | Right | 11 | None | 25.4 | Transient Tinnitus Audio test OK |
| P15 | 48 | M | Ischemic Stroke | Territory of Left Middle Cerebral Artery | Right | 4 | Amateur | 26.6 | Audio test OK |

Table S3.1: Descriptive information of each patient

Fifteen healthy participants were recruited, according to the inclusion criteria, and selected to be appaired in gender to the patient group and in same age range.

| **Healthy** | **Age** | **Gender** | **Musical Background** | **MBEA Average** | **Hearing troubles / Exploration** |
| --- | --- | --- | --- | --- | --- |
| H1 | 24 | M | Amateur | 27.5 | Tinnitus |
| H2 | 37 | M | Professional | 30 | Transient tinnitus |
| H3 | 21 | F | None | 28 | None |
| H4 | 23 | M | None | 27.2 | Tinnitus Audio test OK |
| H5 | 30 | F | Amateur | 27.5 | None |
| H6 | 34 | M | Amateur | 25.7 | Tinnitus Audio test OK |
| H7 | 61 | F | Amateur | 28.7 | None |
| H8 | 32 | F | Amateur | 28.8 | None |
| H9 | 29 | M | Professional | 29.3 | Tinnitus |
| H10 | 46 | F | None | 25.3 | None |
| H11 | 42 | F | None | 24.2 | Audio test OK |
| H12 | 58 | F | None | 26.7 | Audio test OK |
| H13 | 52 | M | None | 23.3 | None |
| H14 | 71 | F | None | 26 | None |
| H15 | 68 | F | Amateur | 24 | Audio test OK |

Table S3.2: Descriptive information of each healthy participant

## S4: Amusia Scores

##
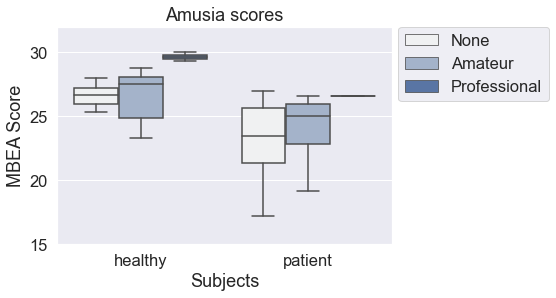


Plot of the amusia score for the healthy and patient participants, considering different levels of music practice: non-musician (“None”), Amateur and Professional. The box of the boxplots represents the limit of the 25th percentile and 75th percentile, the median being indicated inside.

## S5: No Sound Serie


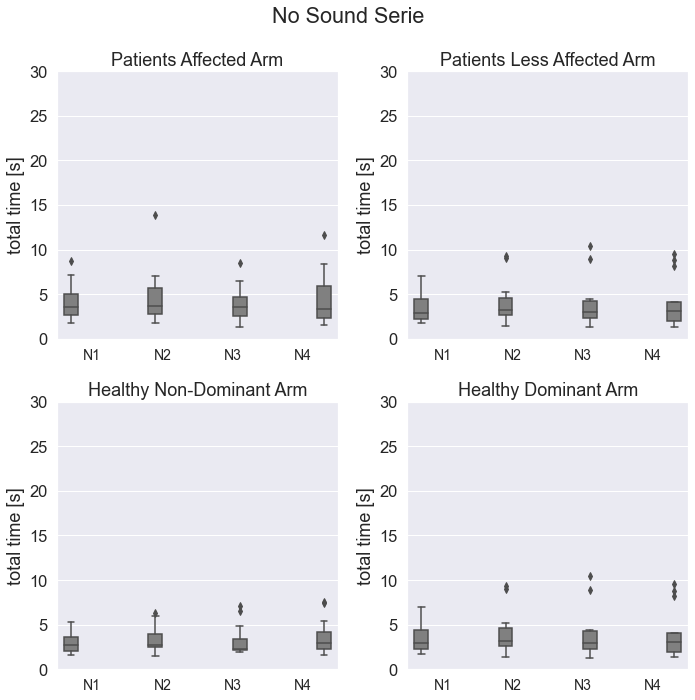


“No sound” series for patients and healthy subjects. The y-axis range [0-30] has been chosen to be identical to figure 4 to facilitate the comparison. The box of the boxplots represents the limit of the 25th percentile and 75th percentile, the median being indicated inside.

## S6: Sonification Categories results


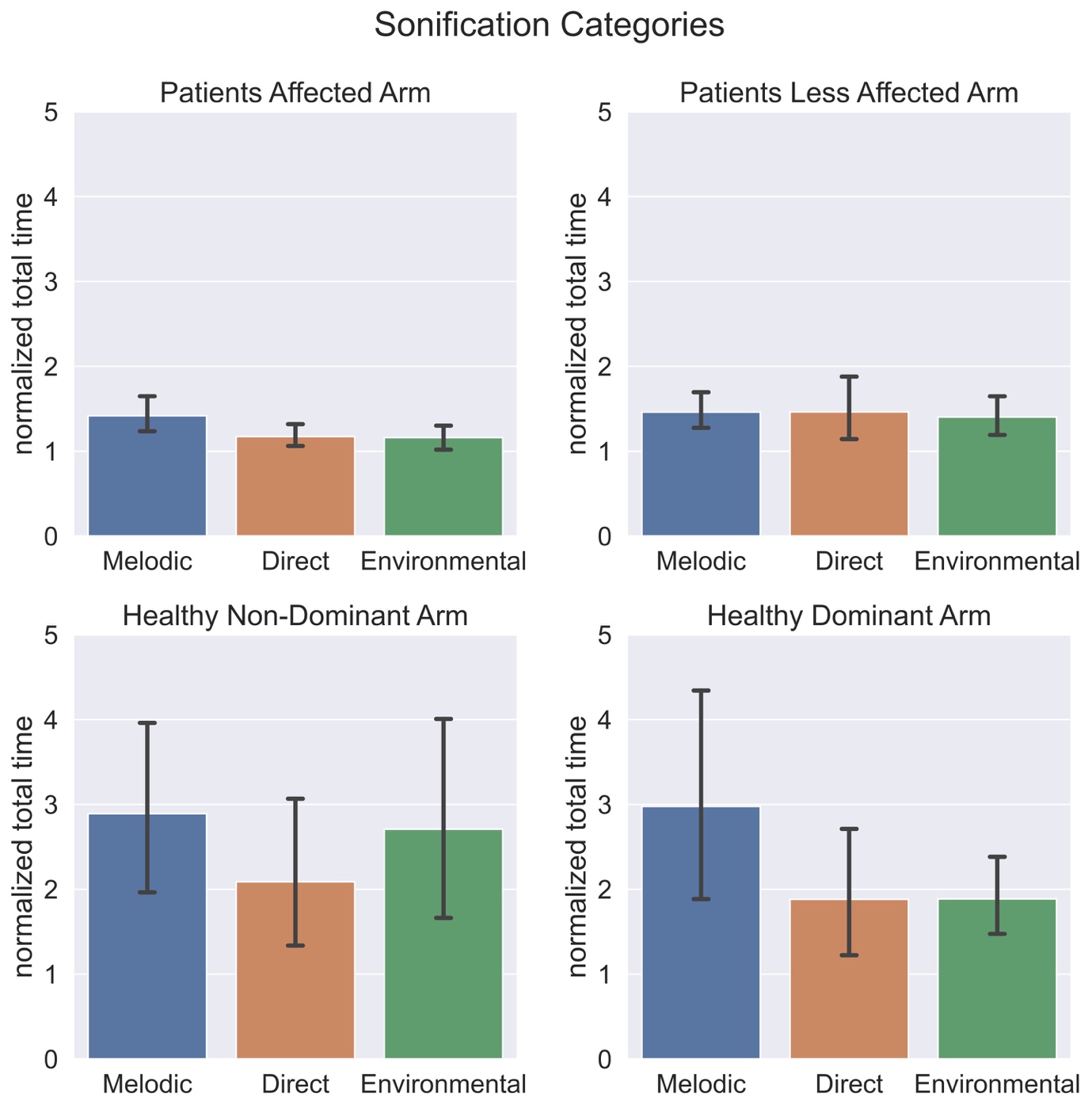


Mean for the patients and healthy participants, for the categories of sound conditions, considering the different arms (affected / less affected for the patients, and non-dominant / dominant for the healthy participants). The error bars correspond to the 95% confidence intervals.

## S7: Sound condition results

The comparative analysis of each type of sound (Friedman’s test) revealed no significant differences. However, there are interestingly several tendencies worth noting on descriptive analysis of repeated measures for each sound condition.


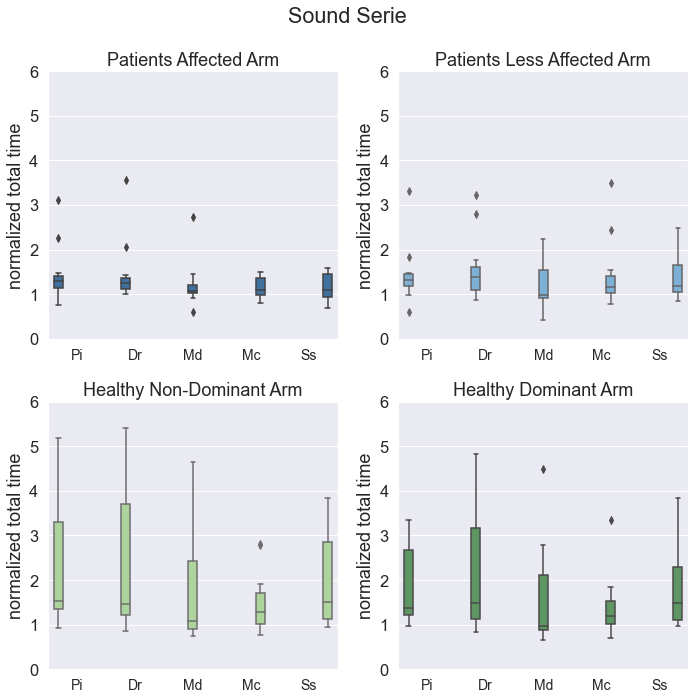


Boxplots of the normalized total time for each sound condition, reported for the Patients and Healthy participants, considering each arm. The box of the boxplots represents the limit of the 25th percentile and 75th percentile, the median being indicated inside.
